# Supplementary material for: Climate Change Impacts on the Potential Distribution Pattern of Osphya (Coleoptera: Melandryidae), an Old but Small Beetle Group Distributed in the Northern Hemisphere
Source: Insects. 2023 May 18;14(5):476. doi: 10.3390/insects14050476 (PMC10231123; doi:10.3390/insects14050476)
Supplement: Supplementary file 1 [file insects-14-00476-s001.zip › insects-2383711-supplementary.pdf]

SUPPLEMENTARY FILES

Table S1. The occurrence records of *Osphya* in constructing model

Species	Distribution	Longitude	Latitude	Source
Osphya bipunctata	Austria	16.2	48.2	GBIF
**(Fabricius, 1775)**	Belgium	4.32827	50.85078	—
	Bosnia	17.68172	44.02904	—
	Bulgaria	23.32166	42.69541	—
	Croatia	15.98554	45.8331	—
	Czech Republic	14.4513	50.07914	—
	Denmark	9.43171	56.11191	—
	France	2.1	49.9	—
	Great Britain	-2.4	51.3	—
	Germany	8	50	—
	Greece	22.93281	40.65901	—
	Hungary	20.5	48.1	—
	Italy	10.5	46.7	—
	Latvia	24.10318	56.95007	—
	The Netherlands	4.31086	52.06358	—
	Norway	10	59.1	—
	Poland	21.08497	52.21973	—

	Romania	26.15526	44.41245	—
	Slovakia	21.27958	48.72323	—
	Sweden	17.6	59.5	—
	Switzerland	7.3	47.4	—
	Ukraine	30.53517	50.45227	—
	Austria	16.2	48.2	—
	Germany	7.1	49.2	—
	Germany	9.7	51.9	—
	Sweden	17.6	59.5	—
	Sweden	16.2	57	—
	Serbia	22.3	42.5	—
	Sweden	16	57	—
	Germany	7.5	49.8	—
	Germany	9.9	51.6	—
	Germany	8.4	49.2	—
	Germany	10.3	50.2	—
	Sweden	15.4	56.3	—
	France	7.6	48.2	—
	Sweden	14.5	56.3	—
	France	2.9	42.5	—
	Norway	10.1	59.2	—

	France	2.9	49.4	—
	UK of Great Britain and Northern Ireland	1.3	52.4	—
	UK of Great Britain and Northern Ireland	-2.2	52.1	—
	France	6.1	47	—
	Sweden	16.6	57.7	—
	France	2.4	49.8	—
	Germany	7.2	50.6	—
	France	3.1	42.5	—
	Germany	8	49.3	—
	Germany	6.2	50	—
	France	5.4	48.5	—
	Germany	11.1	51.3	—
	Germany	7.2	50.5	—
	Germany	6.5	50.6	—
	France	5.6	48.8	—
	Germany	11.3	48.7	—
	Sweden	15.2	56.2	—
	France	5.4	48.3	—
	France	4.8	47.8	—
	France	5.8	45.8	—
	France	5.8	44.1	—

	France	5.8	44.2	—
	Spain	2.5	41.8	—
	France	6.3	46.3	—
	France	5.9	46.1	—
	Norway	9.9	59.2	—
	France	7.5	48.1	—
	France	7.5	48	—
	France	5.5	48.6	—
	Germany	13.6	52.9	—
	Germany	13.9	53.1	—
	Germany	13.8	52.9	—
	Germany	13.9	53.2	—
	Germany	13.8	53	—
	UK of Great Britain and Northern Ireland	0	52.1	—
	France	6.4	44.1	—
	France	5.9	44.3	—
	UK of Great Britain and Northern Ireland	-2.3	51.4	—
	Sweden	16.1	56.9	—
	UK of Great Britain and Northern Ireland	-0.2	52.4	—
	Sweden	15.7	57.6	—
	Sweden	14.5	56.2	—

	France	5.9	44.4	—
	UK of Great Britain and Northern Ireland	0	52.2	—
	Germany	9.3	51.4	—
	Germany	9.1	51.1	—
	France	2.2	42.8	—
	France	3.8	50.2	—
	UK of Great Britain and Northern Ireland	-2	51.8	—
	UK of Great Britain and Northern Ireland	-0.1	52	—
	Germany	9	51.2	—
	France	0.6	42.8	—
	UK of Great Britain and Northern Ireland	-2.3	51.6	—
	UK of Great Britain and Northern Ireland	-0.3	52.6	—
	UK of Great Britain and Northern Ireland	0.5	51.4	—
	UK of Great Britain and Northern Ireland	-0.5	52.6	—
	Austria	16.8	48	—
	UK of Great Britain and Northern Ireland	0.3	51.6	—
	Norway	10.6	59.7	—
	Austria	16.6	47.9	—
	Sweden	16.4	56.9	—
	UK of Great Britain and Northern Ireland	-0.8	52	—
	UK of Great Britain and Northern Ireland	-1.5	51.7	—

	Austria	16.7	48.1	—
	Austria	16.6	47.9	—
	UK of Great Britain and Northern Ireland	-0.3	52.3	—
	UK of Great Britain and Northern Ireland	-1	51.7	—
	UK of Great Britain and Northern Ireland	0.1	51.6	—
	France	-0.6	42.9	—
	Switzerland	7.5	46.3	—
	Austria	14.3	47.9	—
	Austria	16.2	48.1	—
	Austria	16.2	48	—
	Austria	16.4	48.2	—
	Germany	10.6	50.4	—
	France	2.2	42.9	—
	France	-0.6	43	—
	Slovakia	18	48.9	—
	Russia, Chuvash Republic	46.6003	54.9858	Mazurov et al. (2022)
	Russia, Chuvash Republic	47.9125	55.0222	
	Russia, Chuvash Republic	46.7749	55.0091	—
	Russia, Chuvash Republic	46.7203	54.9775	—
	Russia, Saratov Oblast	48.0523	52.4849	—
	Russia, Lipetsk Oblast	38.8942	52.5554	—

	Russia, Lipetsk Oblast	40.0098	52.9792	Egorov et
	Ukraine, Donetsk Donetsk Oblast	37.7029	47.9338	al. (2022)
	Russia, Republic of Moldova	43.1522	54.7275	—
	Russia, Republic of Moldova	43.4371	54.7874	—
	Ukraine, Donetsk Donetsk Oblast	37.7029	47.9338	—
	Russia, Volgograd Oblast	45.6567	50.5724	—
	Russia, Republic of Tatarstan	50.5244	55.6134	—
	Russia, Republic of ufabashkortostan	56.1116	54.5997	—
Osphya aeneipennis	Switzerland	7.9	46.3	GBIF
**Kriechbaumer, 1848**	Switzerland	7.1	46.2	—
	France	6.2	44.1	—
	France	6.6	44.4	—
	France	6.3	43.7	—
	France	6.1	44.6	—
	Switzerland	9.4	46.9	—
	Switzerland	9.2	46.3	—
	France	5.8	43.3	—
	France	5.7	45.2	—
	France	5.8	43.4	—
	France	6.2	44.4	—
	France	2.4	42.6	—

	Switzerland	7.7	46.3	—
	France	5.5	44.4	—
	France	6.4	44	—
	France	5.6	44.9	—
	France	6.6	43.9	—
	France	6.7	44	—
	France	6.3	43.8	—
	France	5.9	43.2	—
	France	6.3	44.1	—
	France	6.8	44.6	—
	Switzerland	7.3	46.3	—
	France	5.6	43.9	—
	France	2.5	42.5	—
	France	6.6	44.3	—
	France	6.8	44.2	—
	Switzerland	8.1	46.3	—
	Switzerland	7.1	46.1	—
	Italy	11.3	46.4	—
	Switzerland	7.9	46.3	—
	Switzerland	10.3	46.8	—
	Switzerland	9.3	46.8	—

	Italy	11.3	46.5	—
	Switzerland	9.5	46.8	—
	Switzerland	7.1	46	—
	Italy	11.2	46.3	—
	Italy	11.7	46.7	—
***Osphya vandalitiae* (Kraatz, 1868)**	Portugal: Faro, Sao Bras de Alportel, Machados.	-7.89209	37.13222	Recalde Irurzun et al. (2017)
	Portugal: Castelhana-aloibre river.	-9.18825	38.71516	—
	Portugal: Bordeira 1.6 Km NE.	-8.86087	37.19622	—
	Portugal: Barranco do Velho (0.5Km S).	-7.93696	37.23770	—
	Portugal: Beja, 15 km of Serpa, Limas river	-7.59764	37.94467	—
	Portugal: Odemira (3.7km NE).	-8.63665	37.59729	—
	Spain: Granada, Guejar Sierra, Vda. LaEstrella.	-3.40457	37.14152	—
	Spain: Cadiz, San Roque, at a pond.	-5.38575	36.21083	—
	Spain: Malaga, Gaucin, from Manilva to Gaucin.	-5.31267	36.51749	—
	Spain: the banks of the Genal river	-5.199563	36.60344	—
	Portugal	-7.6	37.3	GBIF
***Osphya lehnertae* Konvička, 2014**	Greece: Peloponnese, Menalo Mts, 2.2 km	22.171074	37.64855	Konvička

	south southwest of Vytina village.			(2014)
	Greece: Peloponnese, Menalo Mts, Vytina.	22.171074	37.64855	—
	Greece: Peloponnese, Lakonia,Karyes env.	22.44082	37.26141	—
	Greece: Peloponnese, Kalavryta town.	22.14028	37.96050	—
	Greece: Graecia (Patra),5km Nkalavryta.	21.74833	38.23016	—
***Osphya brusteli* Konvička, 2016**	Greece: Western Macedonia Province, Florina env.	21.42324	40.80135	Konvička (2016)
	Greece: Vérno Mts, 2.5 km east southeast of Pisoderi village.	21.268333	40.77389	—
	Greece: Jugoslaviamer, Jakupica mountains, Macedonia centr.	21.40842	41.70836	—
***Osphya cylindromorpha* Abeille, 1896**	Syria	36.19993	35.65025	Nikitsky& Pollock (2008)
	Turkey	36.12423	35.95051	—
***Osphya griseofasciata* Pic, 1921**	Turkey	36.12423	35.95051	Pic (1921b)
***Osphya uniformis* Pic, 1921**	Turkey	36.12423	35.95051	—
***Osphya aerate* Seidlitz, 1898**	Azerbaijan	48.31185	38.69876	Nikitsky& Pollock (2008)

	Armenia	46.41334	39.23411	—
	Iran	48.24487	38.61941	—
***Osphya formosana* Pic, 1927**	China: Taiwan	121.29578	24.99511	Pic (1927a)
***Osphya trilineata* Pic, 1910**	China: Taiwan	121.29578	24.99511	Pic (1910)
***Osphya orientalis* (Lewis, 1895)**	Japan, Miyanoshita	139.06054	35.23929	Lewis (1895)
	Japan, Nikko	139.54738	36.79846	—
***Osphya albofasciata* Champion, 1916**	India: Assam, Patkai Mts.	95.99960	27.00004	Champion (1916)
***Osphya harmandi* Pic, 1926**	India: Sikkim	88.48946	27.21889	Pic (1926)
	India: Darjeeling District	88.27189	27.02923	—
***Osphya dissimilis* Champion, 1922**	India: Uttarakhand, Uttar pradesh	79.06097	30.14047	Champion (1922)
***Osphya nigriventris* Champion, 1920**	India: Uttarakhand, Uttar pradesh	79.06097	30.14047	Champion (1920)
***Osphya nigroapicalis* Pic, 1921**	India.	88.27189	27.02923	Pic (1921a)
***Osphya nilgirica* Champion, 1916**	India: Nilgiri Hills	76.76198	11.37447	Champion (1916)
***Osphya rufa* Pic, 1927b**	Vietnam: Chapa	103.96834	22.50462	Pic (1927b)

Osphya superba **Pic, 1927**	Vietnam: Chapa	103.96834	22.50462	Pic (1927b)
Osphya melina **Champion, 1916**	Myanmar: Tenasserim, Victoria Point.	98.55190	9.99258	Champion (1916)
Osphya essigi **Van Dyke, 1928**	American, Morgan Hill, Santa Clara Co., Calif	-121.64990	37.12581	SBMNH
	USA: Morgan Hill, Santa Clara Co, Calif.	-121.64990	37.12581	—
Osphya lutea **(Horn, 1879)**	USA: Newton.	-118.25111	34.04792	—
	USA: KernSequoia NF, Riverkern.	-118.43283	35.77752	—
	USA: Los Angeles, Pasadena.	-118.14362	34.14781	—
	USA: Los Angeles, Pomona.	-117.76312	34.05376	—
	USA: Los Angeles, Angeles NF, Crystal Lake Rd.	-117.82884	34.32615	—
	USA: Los Angeles, Angeles NF, 4 Km W Lake Hughes.	-118.55842	34.71113	—
	USA: Los Angeles, Angeles NF, LkHughes Rd.	-118.50583	34.64332	—
	USA: Los Angeles, Angeles NF, near Redbox, San Gabriel Mts.	-118.10543	34.25754	—
	USA: Los Angeles, Angeles NF, San Dimas Experimental Forest.	-117.76983	34.16763	—
	USA: Los Angeles, Griffith Park, Griffith	-118.29972	34.13583	—

	Park.			
	USA: Los Angeles, Malibu Ck. SP.	-118.72153	34.09793	—
	USA: Orange, 4 mi E Olive.	-117.78142	33.83342	—
	USA: Riverside, 1 Km S Bundy Cyn, nr. Menifee Valley.	-117.25792	33.61534	—
	USA: Riverside, Devore.	-117.40064	34.21642	—
	USA: Riverside, Strawberry Cyn Rt.	-116.77072	33.70934	—
	USA: Riverside, San Bernardino, NF, Indian Ck.	-116.81733	33.80954	—
	USA: San Bernardino, NF, 1.5 mi S Mt. Baldy Village.	-117.63943	34.24974	—
	USA: San Bernardino, NF, 5 mi SE Hesperia.	-117.23654	34.34234	—
	USA: San Bernardino, NF, Gobblers Knob.	-117.58353	34.31163	—
	USA: San Bernardino, NF, Lake Arrowhead.	-116.98364	34.18901	—
	USA: San Diego, Boulevard.	-116.27283	32.66364	—
	USA: San Diego, Pine Valley.	-116.53423	32.82342	—
	USA: San Diego, River, 7 mi E Lakeside.	-116.80374	32.88323	—
	USA: San Diego.	-117.15643	32.71534	—
	USA: Santa Barbara, Montecito, nr. San Ysidro Ck.	-119.55324	34.49524	—

	USA: Santa Barbara, Foothill Rd.	-119.74363	34.46384	—
	USA: Santa Barbara, San Jose Ck BM.	-119.70272	34.41684	—
	USA: Santa Barbara, Arroyo Hondo Preserve, 25mi.W Sta.Barbara.	-120.14074	34.47843	—
	USA: Santa Barbara, UC Sedgwick Reserve.	-120.03953	34.71324	—
	USA: Tulare, Three Rivers, Ash Mt.	-118.83153	36.49194	—
	USA: Ventura, Howard Creek.	-119.21673	34.54152	—
	USA: Ventura, Ojai.	-119.24192	34.44813	—
	USA: Ventura; Los Padres NF; Upper Sespe Ck.	-119.2863	34.59	—
***Osphya varians* (LeConte, 1866)**	Cananda (Ontario Quebec)	-74.73034	45.04079	—
	United States of America	-94.9	31.3	—
	United States of America	-97.8	30.4	—
	United States of America	-97.4	32.8	—
	United States of America	-97.7	30.4	—
	United States of America	-96.7	32.9	—
	United States of America	-97.6	30.7	—
	United States of America	-97	32.6	—
	United States of America	-80.9	33.5	—
	United States of America	-98.5	33.9	—

	United States of America	-97.4	35.2	—
	United States of America	-77.1	38.9	—
	United States of America	-89.5	43.1	—
	United States of America	-97.7	30.6	—
	United States of America	-97.8	30.2	—
	United States of America	-96.7	33	—
	United States of America	-97.5	30.3	—
	United States of America	-97.2	32.8	—
	United States of America	-81	34.1	—
	United States of America	-97.5	30.2	—
	United States of America	-97.7	32.8	—
	United States of America	-97.3	32.9	—
	United States of America	-97	32.3	—
	United States of America	-97.1	33.1	—
	United States of America	-87.6	33	—
	United States of America	-95.4	36.3	—
	United States of America	-96.8	33.9	—
	United States of America	-98.1	30.3	—
	United States of America	-95.3	39	—
	United States of America	-97.8	30.1	—
	United States of America	-98.8	37.1	—

	United States of America	-98.9	31.6	—
	United States of America	-99.4	37.3	—
	United States of America	-96.2	39.6	—
	United States of America	-95.2	38.8	—
	United States of America	-95	38.8	—
	United States of America	-96.6	39.3	—
	United States of America	-94.8	37.4	—
	United States of America	-99.5	39.4	—
	United States of America	-94.8	37.4	—
	United States of America	-95.4	39.2	—
	United States of America	-95.5	37.3	—
	United States of America	-99.3	37.4	—
	United States of America	-97.3	37.5	—
	United States of America	-95.5	38.9	—
	United States of America	-96.3	29.9	—
	United States of America	-83.9	39.7	—
	United States of America	-82.5	39.5	—
	United States of America	-83.8	39.8	—
***Osphya obscura* Pic, 1937**	Mexico.	-92.51243	16.06642	Pic (1937)
***Osphya pallida* Champion, 1889**	Guatemala: Cerro Zunil, Pantaleon.	-91.49607	14.77672	Champion

				(1889)
***Osphya tuberculiventris* Champion, 1889**	Guatemala: San Gerónimo	-90.24215	15.06085	—
***Osphya* sinensis sp.**	China: Hubei, Shennongjia, Dajiuhu, Luoyanghe	110.13778	31.57722	This study
	China: Hubei, Shennongjia, Dajiuhu, Dongxi	110.12194	31.53944	—

Table S2. Twenty environmental variables in building the initial MaxEnt model

**Class**	**Variables**	**Description**	**Unit**
Bioclimatic variables	BIO1	Annual Mean Temperature	℃
	BIO2	Mean Diurnal Range (Mean of monthly (max temp - min temp))	-
	BIO3	Isothermality (BIO2/BIO7) (×100)	℃
	BIO4	Temperature Seasonality (standard deviation ×100)	℃
	BIO5	Max Temperature of Warmest Month	℃
	BIO6	Min Temperature of Coldest Month	℃
	BIO7	Temperature Annual Range (BIO5-BIO6)	℃
	BIO8	Mean Temperature of Wettest Quarter	℃
	BIO9	Mean Temperature of Driest Quarter	℃
	BIO10	Mean Temperature of Warmest Quarter	℃
	BIO11	Mean Temperature of Coldest Quarter	℃
	BIO12	Annual Precipitation	mm

	BIO13	Precipitation of Wettest Month	mm
	BIO14	Precipitation of Driest Month	mm
	BIO15	Precipitation Seasonality (Coefficient of Variation)	1
	BIO16	Precipitation of Wettest Quarter	mm
	BIO17	Precipitation of Driest Quarter	mm
	BIO18	Precipitation of Warmest Quarter	mm
	BIO19	Precipitation of Coldest Quarter	mm
Topographic variables	ALT	The Elevation	m

Table S3. Multicollinearity test of Pearson correlation analysis for the informative environmental variables

	bio_1	bio_2	bio_3	bio_4	bio_8	bio_9	bio_12	bio_15	bio_17	bio_19	ALT
bio_1	1										
bio_2	.573**	1									
bio_3	.623**	.585**	1								
bio_4	-.171**	.225**	-.630**	1							
bio_8	.522**	.203**	-.023	.335**	1						
bio_9	.563**	.399**	.688**	-.523**	-.291**	1					
bio_12	.099	-.218**	.021	-.162**	.138*	-.038	1				
bio_15	.548**	.528**	.702**	-.315**	.064	.550**	.082	1			
bio_17	-.446**	-.445**	-.508**	.156**	-.116	-.409**	.404**	-.769**	1		
bio_19	.000	.023	.201**	-.279**	-.519**	.471**	.252**	.210**	.130*	1	

ALT	-.347**	.025	.098	-.152*	-.501**	.136*	.388**	.203**	.179**	.329**	1

Note: **means significant correlation at 0.01 level (bilateral). *means significant correlation at 0.05 level (bilateral).

Table S4. The performance of MaxEnt models under different climate scenarios.

	LGM	MID	Current	2050s RCP4.5	2050s RCP8.5	2070s RCP4.5	2070s RCP8.5
AUC	0.966	0.967	0.967	0.969	0.966	0.966	0.968
TSS	0.872	0.941	0.899	0.876	0.868	0.868	0.869

Table S5. The potential distribution area of *Osphya* under different climate scenarios (Units 10^4 Km^2).

Habitat suitability	LGM	MID	Current	2050s RCP4.5	2050s RCP8.5	2070s RCP4.5	2070s RCP8.5
Suitable (0.2-1.0)	645.98	1015.18	1049.21	1051.73	1257.49	1163.56	1196.31
Low (0.2-0.4)	491.28	830.60	850.74	880.69	1018.02	954.08	981.43
General (0.4-0.6)	110.96	161.92	164.82	155.24	214.59	187.89	195.09
Medium (0.6-0.8)	39.17	22.25	31.32	14.58	22.71	20.11	17.79
High (0.8-1.0)	4.56	0.42	2.33	1.22	2.18	1.48	2.00

Table S6. Distribution changes of *Osphya* under the different climate scenarios (units in Km^2). The percentage of the potential area compared to the total study area is shown in brackets.

Dynamic changes	LGM	MID	2050s RCP4.5	2050s RCP8.5	2070s RCP4.5	2070s RCP8.5
Range expansion	4953614.34 (3.63%)	1932449.41 (1.42%)	1520277.75 (1.12%)	3044638.52 (2.23%)	2255237.95 (1.65%)	2965088.30 (2.18%)
No occupancy	125024390.66 (91.68%)	124279979.85 (91.14%)	124352006.29 (91.19%)	122827645.51 (90.07%)	123617046.08 (90.65%)	122907195.73 (90.13%)
No change	5540071.64 (4.06%)	8561267.15 (6.28%)	8992110.64 (6.59%)	9528103.55 (6.99%)	9381756.84 (6.88%)	8991927.13 (6.59%)
Range contraction	845691.29 (0.63%)	1592304.18 (1.16%)	1501605.92 (1.10%)	965613.02 (0.71%)	1111959.72 (0.82%)	1501789.43 (1.10%)

Table S7. Percent contribution and permutation importance of environmental variables in building MaxEnt model

Class	Variables	Description	Unit	Percent contribution	Permutation importance
Bioclimatic variables	bio_1	Annual Mean Temperature	℃	19.3	22.1
	bio_2	Mean Diurnal Range (Mean of monthly (max temp - min temp))	℃	1.9	1.4
	bio_3	Isothermality (BIO2/BIO7) (×100)	-	16.3	9.5
	bio_4	Temperature Seasonality (standard deviation ×100)	℃	14	18.6
	bio_8	Mean Temperature of Wettest Quarter	℃	1	9.5
	bio_9	Mean Temperature of Driest Quarter	℃	1.9	3.5
	bio_12	Annual Precipitation	mm	6.4	14.8
	bio_15	Precipitation Seasonality (Coefficient of Variation)	1	1.5	6.4
	bio_17	Precipitation of Driest Quarter	mm	3.6	3.3
	bio_19	Precipitation of Coldest Quarter	mm	32.6	9.1
Topographic variables	ALT	The Elevation	m	1.2	1.8

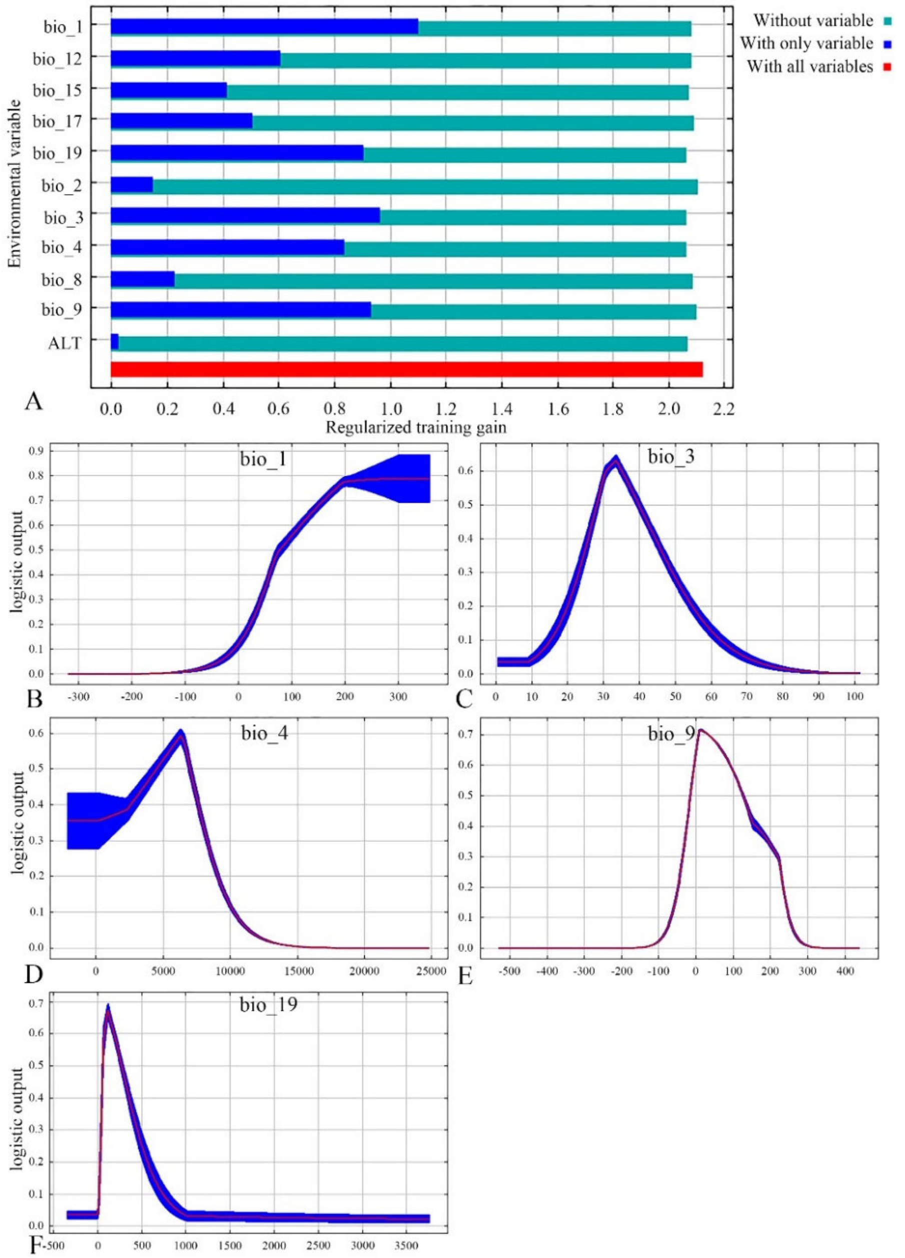

Figure S1. (**A**) The results of Jackknife of regularized training gain for *Osphya*; (**B–F**) response curves of the five most important environmental variables affecting distribution of *Osphya*: (**B**) annual mean temperature; (**C**) isothermality; (**D**) temperature seasonality; (**E**) mean temperature of driest quarter; (**F**) precipitation of coldest quarter. Blue margins represent ± SD calculated over 10 replicates.
